# Distinct profile of CD34^+^ cells and plasma-derived extracellular vesicles from triple-negative patients with Myelofibrosis reveals potential markers of aggressive disease

**DOI:** 10.1186/s13046-020-01776-8

**Published:** 2021-02-01

**Authors:** Dorian Forte, Martina Barone, Cristina Morsiani, Giorgia Simonetti, Francesco Fabbri, Samantha Bruno, Erika Bandini, Daria Sollazzo, Salvatore Collura, Maria Chiara Deregibus, Giuseppe Auteri, Emanuela Ottaviani, Nicola Vianelli, Giovanni Camussi, Claudio Franceschi, Miriam Capri, Francesca Palandri, Michele Cavo, Lucia Catani

**Affiliations:** 1 Azienda Ospedaliero-Universitaria di Bologna, via Albertoni 15, Bologna, Italy; 2 Istituto di Ematologia “Seràgnoli”, Dipartimento di Medicina Specialistica, Diagnostica e Sperimentale, Università degli Studi, Bologna, Italy; 3 Department of Experimental, Diagnostic and Specialty Medicine (DIMES), University of Bologna, Bologna, Italy; 4 Istituto Scientifico Romagnolo per lo Studio e la Cura dei Tumori (IRST) IRCCS, Meldola, Italy; 5 Department of Internal Medicine, Centre for Molecular Biotechnology and Centre for Research in Experimental Medicine, Torino, Italy; 6 Laboratory of Systems Medicine of Healthy Aging and Department of Applied Mathematics, Lobachevsky University, Nizhny Novgorod, Russia

**Keywords:** Myelofibrosis, Extracellular vesicles, Inflammation, microRNAs

## Abstract

**Background:**

Myelofibrosis (MF) is a clonal disorder of hemopoietic stem/progenitor cells (HSPCs) with high prevalence in elderly patients and mutations in three driver genes (*JAK2*, *MPL*, or *CALR*). Around 10–15% of patients are triple-negative (TN) for the three driver mutations and display significantly worse survival. Circulating extracellular vesicles (EVs) play a role in intercellular signaling and are increased in inflammation and cancer. To identify a biomolecular signature of TN patients, we comparatively evaluated the circulating HSPCs and their functional interplay with the microenvironment focusing on EV analysis.

**Methods:**

Peripheral blood was collected from MF patients (*n* = 29; *JAK2*^*V617F*^ mutation, *n* = 23; TN, *n* = 6) and healthy donors (HD, *n* = 10). Immunomagnetically isolated CD34^+^ cells were characterized by gene expression profiling analysis (GEP), survival, migration, and clonogenic ability. EVs were purified from platelet-poor plasma by ultracentrifugation, quantified using the Nanosight technology and phenotypically characterized by flow cytometry together with microRNA expression. Migration and survival of CD34^+^ cells from patients were also analyzed after in vitro treatments with selected inflammatory factors, i.e. (Interleukin (IL)-1β, Tumor Necrosis Factor (TNF)-α, IL6) or after co-culture with EVs from MF patients/HD.

**Results:**

The absolute numbers of circulating CD34^+^ cells were massively increased in TN patients. We found that TN CD34^+^ cells show in vitro defective functions and are unresponsive to the inflammatory microenvironment. Of note, the plasma levels of crucial inflammatory cytokines are mostly within the normal range in TN patients. Compared to JAK2^V617F^-mutated patients, the GEP of TN CD34^+^ cells revealed distinct signatures in key pathways such as survival, cell adhesion, and inflammation. Importantly, we observed the presence of mitochondrial components within plasma EVs and a distinct phenotype in TN-derived EVs compared to the JAK2^V617F^-mutated MF patients and HD counterparts. Notably, TN EVs promoted the survival of TN CD34^+^ cells. Along with a specific microRNA signature, the circulating EVs from TN patients are enriched with miR-361-5p.

**Conclusions:**

Distinct EV-driven signals from the microenvironment are capable to promote the TN malignant hemopoiesis and their further investigation paves the way toward novel therapeutic approaches for rare MF.

**Supplementary Information:**

The online version contains supplementary material available at 10.1186/s13046-020-01776-8.

## Background

Myelofibrosis (MF) is a chronic myeloproliferative neoplasm (MPN) that can be diagnosed as a primary disease (PMF) or secondary to polycythemia vera or essential thrombocythemia (PPV/PET-MF, also known as SMF). MF predominantly affects elderly patients with more than 65% of diagnosis occurring after 65 years of age [1] and is clinically characterized by debilitating systemic symptoms, progressive splenomegaly, cytopenias, and overall reduced survival, mainly due to disease progression and leukemic transformation [2–4].

The molecular pathogenesis of MF relates to mutations in three “driver” genes (namely: *JAK2*, *CALR*, *MPL*) with around 60% of patients showing the *JAK2*^*V617F*^ mutation. Interestingly, 10% of patients are unmutated for the *JAK2*, *CALR,* and *MPL* genes and are classified as triple-negative (TN) [4]. Irrespective of “driver” mutations, a hyper-activation of the JAK-STAT pathway is observed in all MF patients [5]. The molecular basis of TN remains mostly unknown, although a high molecular complexity has been previously described [6] and rare, alternative, somatic mutations in both *JAK2* exon 14 and *MPL* exon 10 have been previously annotated [7, 8]. TN MF is associated with an aggressive clinical behavior characterized by a higher risk of developing anemia and thrombocytopenia, poorer outcomes in comparison with patients affected by the other MF molecular subtypes and a high rate of leukemic transformation [9, 10].

Besides molecular alterations, a state of chronic inflammation involving the malignant hemopoietic stem/progenitor cells (HSPCs) and the non-malignant/malignant microenvironment has been indicated as the main contributor in MF initiation/clonal evolution [11, 12]. Abnormal expression and activity of several cytokines involved in inflammation and immunoregulation are described in MF [13, 14] and correlate with more severe marrow fibrosis [15], worsening systemic symptoms [16], and decreased survival [17]. Importantly, this chronic inflammation may allow the neoplastic clone to gain a selective advantage [18, 19] and the potential role of the interaction between the inflammaging process [20] and niche effect has recently been raised up [21].

Extracellular vesicles (EVs) have emerged as crucial actors in intercellular communication. Involved in a myriad of biological processes, including regulation of immunity and inflammation [22, 23], they are released from a variety of cell types exerting pleiotropic effects. EVs present antigens and contain constituents from the cell of origin including microRNAs (miRNAs), transcription factors, mitochondria, nucleic acid, and microenvironmental signals that may contribute to the propagation of inflammation [24–26]. Of interest, both damaged and functional mitochondria may be carried by EVs and they exert a role in immune regulation [27–29]. Various hematological malignancies including MPN have been associated with increased numbers of circulating EVs [30–33]. To date, the role of circulating EVs is still elusive in overall MF patients and their characterization remains fully obscure in the TN subclass.

In particular, the aggressive clinical behavior of TN MF patients is currently without any biological explanation and it is currently unclear the role of the HSPCs and/or the inflammatory microenvironment of TN MF patients. Here, we aim to identify a distinct signature of circulating CD34^+^ cells, cytokines, and EVs isolated from TN MF patients by comparatively evaluating the *JAK2*^*V617F*^-mutated counterparts.

## Materials and methods

### Patients

Peripheral blood (PB) was obtained from and MF patients (*n* = 29) and age-matched healthy donors (HD; *n* = 10). The clinical/laboratory characteristics of the patient cohorts are shown in Additional file 1: Table S1. In 15 pts., who received previous treatment (Hydroxyurea/Ruxolitinib), therapies had been discontinued for at least 3 months before sample collection.

### CD34^+^ cells isolation

PB, anticoagulated with ethylenediaminetetraacetic acid (EDTA), was obtained from patients/controls. Mononuclear cells (MNC) were separated from MF and cord blood (CB) samples by stratification on Lympholyte-H 1.077 g/cm3 gradient (Gibco-Invitrogen, Milan, Italy), followed by red blood cell lysis for 15 min at 4 °C. MNCs were then processed on magnetic columns for CD34^+^ cell isolation (mean purity 94% ± 5%) (MACS CD34 Isolation kit; Miltenyi Biotech, Bologna, Italy), as previously described [19].

### Functional characterization of CD34^+^ cells

Immunomagnetically isolated CD34^+^ cells from MF patients or CB units were maintained in RPMI 1640 with 10% fetal bovine serum (FBS) with or without IL-1β (10 ng/mL, Thermo Scientific Pierce Biotechnology, Rockford, IL, USA), TNF-α (100 ng/mL, Thermo Scientific), IL-6 (10 ng/mL, Thermo Scientific), alone or in combination, for 24 h. In vitro survival was analyzed as previously described by apoptotic assay [19, 34].

### Clonogenic assay of CD34^+^ cells

MF/CB-derived CD34^+^ cells were cultured in vitro to achieve hematopoietic cell differentiation and the formation of multi-lineage colony-forming units (CFU-Cs), including colony forming unit-granulocyte macrophage (CFU-GM) and Burst Forming Unit-erythroid (BFU-E) in the presence or the absence of IL-1β (1 ng/mL), TNF-α (100 ng/mL), IL-6 (10 ng/mL), alone or in combination, as previously described [19, 34].

### Migration assay of CD34^+^ cells

Migration of MF/CB purified CD34^+^ cells were assayed towards a CXCL12 gradient (150 ng/ml, R&D) in transwell chambers (diameter 6.5 mm, pore size 8 μm; Costar; Corning), as previously described [19, 34]. Specifically, 50 μl of RPMI 1640 plus 10% FBS containing 0,5 × 10^5^ cells were added to the upper chamber and 150 μl of medium with or without CXCL12 ± IL-1β (1 ng/mL], TNF-α (100 ng/mL), IL-6 (10 ng/mL) (alone or in combination) were added to the bottom chamber.

### The phenotype of circulating CD34^+^ cells

The phenotype of circulating CD34^+^ cells was evaluated in PB from MF patients and in CB samples by conventional immunofluorescence, as previously described [19, 34]. A minimum of 1 × 10^4^ CD34^+^ cells was acquired by flow cytometer BD Accuri C6 (Becton Dickinson). The analysis was performed excluding cellular debris in an SSC/FSC dot plot. The percentage of positive cells was calculated by subtracting the value of the appropriate isotype controls. The absolute number of positive cells/mL was calculated as follows: the percentage of positive cells × White Blood Cell count/100.

### Gene expression profiling (GEP) of circulating CD34^+^ cells

GEP was performed on RNA samples of immunomagnetically isolated CD34^+^ cells from MF patients using GeneChip Human Transcriptome Array 2.0 (Thermo Fisher Scientific), according to the manufacturer’s recommendations. Data quality control and normalization (signal space transformation robust multiple-array average, sst-RMA) and supervised analysis were carried out Transcriptome Analysis Console v4.0 software (Thermo Fisher Scientific). Fold-change absolute value ≥2 and *p* ≤ 0.05 were used as a cut-off. The resulting genes were selected for functional annotation clustering, that was performed using David Bioinformatics Resources v6.8 (National Institute of Allergy and Infectious Diseases, NIH) [35]. Gene set enrichment analysis (GSEA) was performed with GSEA v3.0 software (Broad Institute) [36].

### Collection of blood samples and isolation/enumeration of circulating EVs

Blood samples were collected into K2EDTA-containing collection tubes (Vacutainer® tubes, Becton Dickinson), plasma and EVs were prepared as previously described with minor modifications [30, 37]. Briefly, platelet-poor plasma (PPP) was obtained (within 2 h from blood collection) after two consecutive centrifugations at 2500 g for 15 min at room temperature. The supernatant was subsequently ultracentrifuged at 100,000×g for 2 h at 4 °C using Optima L-90 K ultracentrifuge (Beckman Coulter) equipped with Type 50.2 Ti rotor. After centrifugation, pelleted EVs were resuspended and washed with Dulbecco’s PBS (DPBS; Sigma Aldrich). Finally, EVs were resuspended in saline buffer solution with 1% DMSO and stored at − 80 °C and/or used for further experiments. Isolated EVs were analyzed by nanoparticle tracking analysis (NTA), using the NanoSight LM10 system (NanoSight Ltd., Amesbury, UK), equipped with a 405 nm laser and with the NTA 2.3 analytic software, to define their dimension and profile.

### The phenotype of the isolated EVs

To phenotype EVs isolated from patients/HD, the MACSPlex Exosome Kit (Miltenyi Biotec GmbH, Bergisch Gladbach, Germany) was utilized. It provides the detection of 37 surface epitopes plus 2 isotype controls as for manufacturer’s instructions. This approach allows semi-quantitative analysis of differential surface epitopes. The proportion of megakaryocyte (MK)- and platelets (PLT)-EVs in the isolated EVs samples was analyzed by flow cytometry as previously described [30] using CytoFLEX (Beckman Coulter).

Where indicated, MitoTracker™ Red CMXRos (Thermo Fisher Scientific, Waltham, MA, USA) was used to stain mitochondria/mitochondria components in plasma EVs following the manufacturer’s instructions and the geometric mean of fluorescence intensity (MFI) was determined by flow cytometry.

Appropriate controls were used: single color stains, sample dilution of EVs in double-filtered PBS or their buffer, unstained EVs were used to determine the fluorescence background as well as the buffer with antibody/dye. Antibody/dye filtration was performed before staining using Ultrafree®-MC/Durapore®-PVDF centrifugal filter units.

### Transmission electron microscopy (TEM) of the isolated EVs

TEM was performed on EVs isolated by ultracentrifugation, placed on 200 mesh nickel formvar carbon-coated grids (Electron Microscopy Science, Hatfield, PA, USA) and left to adhere for 20 min. The grids were then incubated with 2.5% glutaraldehyde containing 2% sucrose and after washings in distilled water the EVs were negatively stained with NanoVan (Nanoprobes, Yaphank, NK, USA) and observed using a Jeol JEM 1010 electron microscope (Jeol, Tokyo, Japan).

### Western blot analysis

EV protein extracts were separated by Sodium Dodecyl Sulphate-PolyAcrylamide Gel Electrophoresis (SDS-PAGE, Bio-Rad) and transferred onto nitrocellulose membranes.

Membranes were incubated overnight with the following antibodies: rabbit anti-TOMM20 (AbCam, Cambridge, UK; ab56783) and goat anti-ß-tubulin (Santa Cruz Biotechnology) as control. Horseradish peroxidase (HRP) conjugated anti-rabbit Immunoglobuluin (Ig) G (GE Healthcare) and anti-goat IgG (Santa Cruz) were used as secondary antibodies. Enhanced chemiluminescence Prime (ECL™) reaction kit (GE Healthcare, Amersham, U.K.) was utilized for detection by ChemiDoc XRS+ System (Bio Rad) and Image J software was applied to perform signal quantification.

### Analysis of the miRNAs cargo of the isolated EVs

miRNA expression of isolated EVs (10^9^) from patients/HD was investigated after RNA extraction with the miRNeasy Micro kit (Qiagen, Milan, Italy) and TaqManTM Array Human MicroRNA A and B Cards (Applied Biosystems by Life Technologies, NY, USA) according to manufacturer’s protocol. This discovery phase was assessed on 3 HD and 6 MF. In the latter case, patients were equally divided in *JAK2*^V617F^-mutated and TN. To discover significantly different expressed miRNAs, RT-qPCR validation assay was performed, as previously described, in the same samples used for the profiling and in an enlarged cohort of 10 HD, 6 TN and 10 *JAK2*^V617F^-mutated patients in total. Data were normalized with cel-miR-39 spiked-in at lysis step of RNA extraction. MiRNAs’ targets were investigated via KEGG analysis, using the mirPath software with DIANA tools [38]. This analysis is based on the already validated targets reported in the Tarbase database.

### EV co-culture studies

Circulating CD34^+^ cells isolated from CB or MF patients were seeded in 96 wells plate and co-cultured over-night with plasma-derived EVs from HD or MF patients, respectively. EV concentration was calculated using Nanosight (Malvern) and EV concentration for co-cultures was determined by dose titration. Cells were then collected, counted, and stained with Annexin V and PI to detect the apoptotic rate by flow cytometry, as above described.

### Plasma levels measurement of selected circulating cytokines

We measured the cytokine plasma levels of patients/HD by ELISA, according to the manufacturer’s instructions. PPP was obtained (within 2 h from blood collection) after two consecutive centrifugations at 2500 g for 15 min at room temperature. The plasma was then collected and stored at − 80 °C until quantification. In particular, the Human Thrombopoietin Quantikine ELISA Kit was provided from R&D Systems (Minneapolis, Minnesota, USA) and CXCL12 ELISA kit from Krishgen ByoSistems (Ashley CT, Whittier, CA, USA). The CiraplexTM immunoassay kit / Human 9-Plex Array (Aushon BioSystems, Billerica, MA, USA) was used for the measurement of various cytokines including IL-1β and TNF-α.

### Mutation analysis

*JAK2*^V617F^ allele-burden was assessed in granulocyte DNA with ipsogen JAK2 MutaQuant Kit (Qiagen, Marseille, France) 505 on 7900 HT Fast Real-Time PCR System (Applied Biosystem, Monza, Italy). CALR exon 9 sequencing was performed by Next Generation Sequencing (NGS) approach with GS Junior (Roche-454 platform; Roche Diagnostics, Monza, Italy); analysis was performed with AVA Software (GRCh38 as referenced). Rare CALR mutations identified by NGS were confirmed by Sanger sequencing. MPL mutations were investigated by ipsogen MPLW515K/L MutaScreen Kit (Qiagen) and by Sanger sequencing (for MPLS505N and other secondary exons 10 mutations).

### Statistical analysis

Numerical variables have been summarized by their median and range, and categorical variables by count and relative frequency (%) of each category. Comparisons of quantitative variables between groups of patients were carried out by the nonparametric Wilcoxon rank-sum test. The differences between the groups were analyzed with Mann Whitney, Kruskal Wallis, one-way ANOVA tests as appropriate. Since miRNA fold change values and cytokines values were not normally distributed as computed by Shapiro-Wilk test, we performed Mann Whitney and Kruskal Wallis as non-parametric tests. All *p* values were considered as statistically significant when ≤0.05. Statistical analyses were performed using Graphpad (Graphpad Software Inc., La Jolla, CA) and SPSS software (PASW Statistics for Windows, Version 18.0. Chicago, IL).

## Results

### Circulating CD34^+^ cells of TN patients are functionally defective

MF patients (*n* = 29) and their demographics and clinical-laboratocharacteristics are shown in Table S1. The median age of MF patients was 72 years (range 43–84), 45% were of the female gender. The J*AK2*^*V617F*^ mutations were present in 23 (80%) patients and 6 (20%) were TN. Within the classes, TN patients had significantly higher lymphocyte count compared to *JAK2*^*V617F*^-positive patients (*p =* 0.04).

**Fig. 1:**
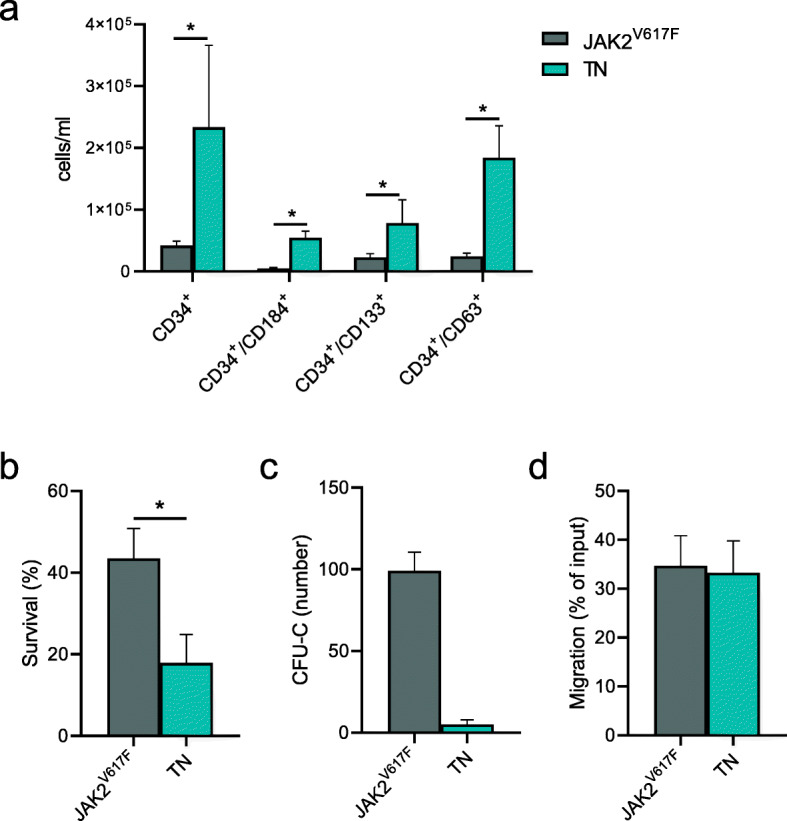
Phenotype and function of circulating CD34^+^ cells. **a** The absolute number of MF patients subdivided into *JAK2*^*V617F*^-mutated (grey column, *n* = 18) or TN (blue column, *n* = 6) groups. CD34^+^ cells co-expressing the CD184, CD133, CD63 antigens are shown. Percentage of Annexin V/PI negative (survival) CD34^+^ cells (**b**), CFU-C counts (**c**), and migration as input (**d**) of cells cultured *in vitro* over-night. All data are presented as mean ± SEM (*p ≤ 0.05)

To investigate the ex vivo behavior of HSPCs, we firstly compared the number, phenotype, and function of CD34^+^ cells isolated from PB of TN and *JAK2*^*V617F*^ mutated patients. We found a 5.5-fold increase in the absolute numbers of circulating CD34^+^ cells from TN (*p =* 0.01). In addition, CD34^+^ cells displayed a higher co-expression of markers such as CD184 (*p =* 0.03), CD133 (*p =* 0.04) and CD63 (*p =* 0.03; Fig. 1a).

To investigate the functionality of circulating CD34^+^ cells, we performed in vitro experiments seeding the cells in overnight cultures in the absence of any stimuli. As shown in Fig. 1b, a marked decrease in the survival rate of TN-derived CD34^+^ cells was observed as compared to the *JAK2*^*V617F*^-mutated counterparts. Again, when we tested the colony-forming capacity (CFU-C), we found the reduced clonogenic output of TN-derived CD34^+^ cells, albeit not statistically significant (Fig. 1c). Conversely, no differences were observed between the two groups of patients when migration ability toward a CXCL12 gradient was tested (Fig. 1d).

### Gene expression profiling (GEP) of PB CD34^+^ cells from MF patients reveals distinct signatures according to their mutational status

To elucidate whether the quanti-qualitative differences between PB CD34^+^ cells from TN and *JAK2*^*V617F*^-mutated patients are due to intrinsic factors, we performed the GEP on circulating CD34^+^ cells isolated from the two groups of patients. Overall, 151 genes were differentially expressed between the two cohorts (34 upregulated and 117 downregulated genes).

**Fig. 2:**
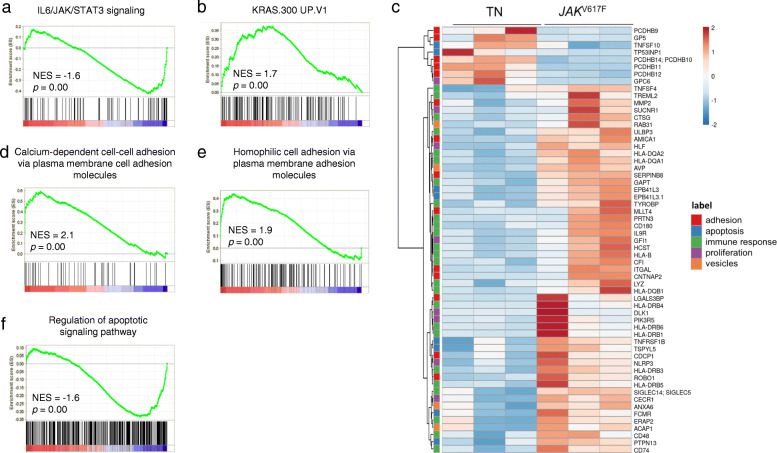
The transcriptional program of CD34^+^ cells differs between TN and *JAK2*^V617F^ MF patients. Signatures IL6/JAK/STAT3 signaling (**a**) and KRAS (**b**) enriched in *JAK2*^V617F^-mutated and TN patients, respectively (GSEA). **c** Gene expression differences in cell adhesion-, apoptosis-, immune response-, proliferation- and vesicle-related genes between *JAK2*^V617F^-mutated (*n* = 3) and TN (*n* = 3) patients. Each column represents a different patient. Data were standardized through a z-score transform; color changes within a row indicate expression levels relative to the mean and rescaled on the transcript standard deviation. Genes are ranked according to average linkage hierarchical clustering. **d-e** Cell adhesion-related signatures in TN, identified by GSEA and enriched in TN. **f** Apoptosis-related signatures enriched in the *JAK2*^V617F^ cohorts. (NES: normalized enrichment score; TN: triple-negative)

A signature of IL6-JAK-STAT3 signaling was significantly enriched in *JAK2*^*V617F*^-mutant compared with the TN CD34^+^ cells, indicating a preferential or enforced activation of the pathway in this cohort (Fig. 2a). Conversely, TN CD34^+^ cells were enriched for a signature of KRAS pathway activation, suggesting alternative oncogenic signaling supporting malignant cell growth (Fig. 2b).

Additional differentially expressed transcripts were enriched for genes involved in antigen processing/presentation, regulation of immune response, inflammation, and vesicles (Additional file 1: Table S2). Of interest, among the inflammation-related genes, *MMP2, CD180, ROBO1, CD48, IL9-R, NLRP3* were significantly downregulated in TN patients as compared to the *JAK2*^*V617F*^ counterparts (Fig. 2c).

Furthermore, 5 adhesion-related genes were upregulated (*PCDHB14*,* PCDHB11*,* PCDHB12, PCDHB9*, and* GP5*) and 9 were downregulated (*CNTNAP2, ITGAL, LGALS3BP, MMP2, AMICA1, ROBO1, CDCP1, MLLT4, SERPINB8*) in TN CD34^+^ cells (Fig. 2c). Among apoptosis-related genes, 2 pro-apoptotic genes (*TNFSF10* and *TP53INP1*) were upregulated, and 4 antiapoptotic (*TSPYL5, FCMR, PTPN13*, *TNFRSF1B*) were downregulated in TN-derived CD34^+^ cells (Fig. 2c). Pathway analysis revealed that several altered genes were involved in cell adhesion, with enrichment of signatures of homophilic cell adhesion and calcium-dependent cell-cell adhesion via plasma membrane adhesion molecules in TN MF (Fig. 2d-e). However, CD34^+^ cells from *JAK2*^*V617F*^-mutated patients were enriched for gene signatures of apoptosis regulation (Fig. 2f). Genes involved in proliferation (*HLF, DLK1, GFI-1, CECR1, NLRP3, PIK3R5, SUCNR1*) were also downregulated.

### The plasma levels of crucial inflammatory cytokines are mostly within the normal range in TN patients

**Fig. 3:**
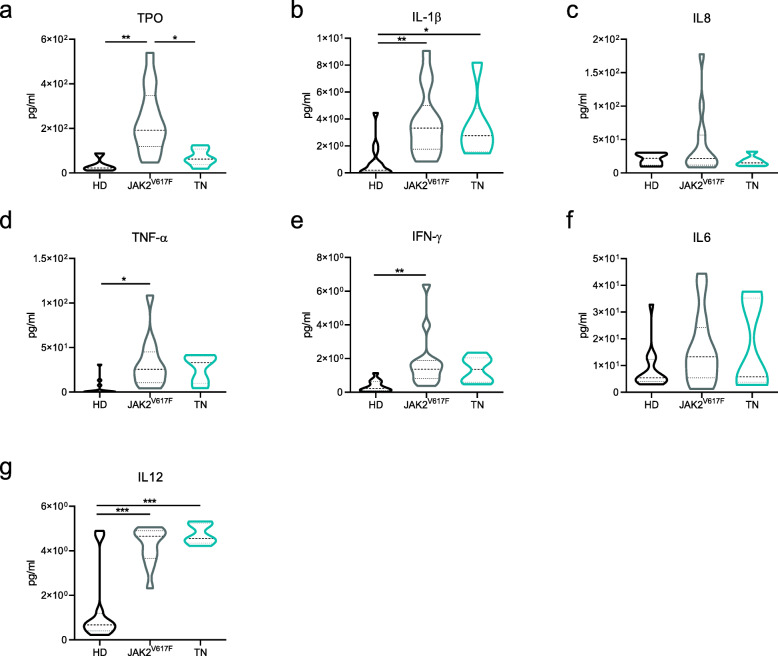
Plasma levels of TPO and pro-inflammatory cytokines in MF patients according to mutational status. Violin plots of TPO (**a**), IL-1β (**b**), IL8 (**c**), TNF-α (**d**), IFN-γ (**e**), IL6 (**f**), and IL12 (**g**) plasma levels in MF patients (*JAK2*^V617F^, *n* = 14; TN, *n* = 6) and HD (*n* = 5-10) measured by ELISA. All data are presented as mean ± SEM (*p ≤ 0.05; **p ≤ 0.01; ***p ≤ 0.001)

To characterize the inflammatory microenvironment of TN patients, we analyzed the plasma concentration of key inflammatory cytokines and TPO. Compared to HD, only IL-1β and IL-12 plasma levels were significantly increased in TN patients (*p* < 0.05; Fig. 3b-g). Conversely, the plasma levels of TPO (*p* < 0.01), IL-1β (*p* < 0.01), TNF-α (*p* < 0.05), IFN-γ (*p* < 0.01), and IL-12 (*p* < 0.001) were significantly increased in the *JAK2*^*V617F*^-mutated patients compared to HD. The plasma levels of TPO were significantly decreased in TN patients compared to *JAK2*^*V617F*^-mutated patients (*p* < 0.05). Interestingly, TPO levels were 2-fold increased in overall female MF patients compared to males (mean: 242 ± 47.16 vs 118 ± 38.85, respectively; *p =* 0.02, data not shown).

### Circulating CD34^+^ cells from TN patients are functionally insensitive to pro-inflammatory stimuli

**Fig. 4:**
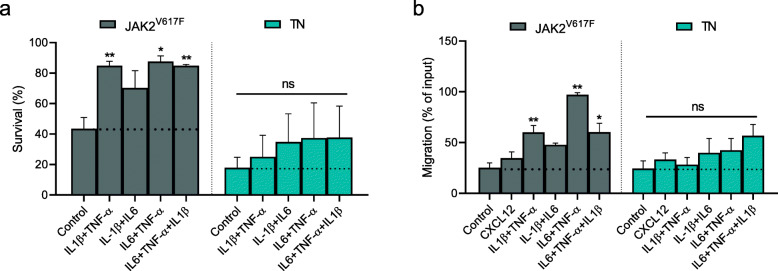
Survival and migration of CD34^+^ cells from TN patients are not increased by pro-inflammatory cytokines. **a** CD34^+^ cells from JAK2^V617F^ (*n* = 4) or TN (*n* = 4) patients were in vitro treated with combined pro-inflammatory factors such as IL-1β, TNF-α, and IL6 and the fold-change of cell viability was assessed after Annexin V/PI staining, as described in Methods. Dot lines were used to mark control samples without any treatment, as fold-change = 1. **b** Spontaneous migration and toward CXCL12, alone or in combination with pro-inflammatory cytokines, were observed in *JAK2*^V617F^-derived (*n* = 4) CD34^+^ cells as compared with the TN (*n* = 4) counterparts. Results are expressed as mean percentages ± SEM of input (*p ≤ 0.05; **p ≤ 0.01)

We then sought to assess whether the in vitro functional defects of CD34^+^ cells from TN patients might be due to abnormal response to the inflammatory signals of the microenvironment. For this purpose, we tested the effects of selected inflammatory cytokines (alone or in combination) on the in vitro functional behavior of PB CD34^+^ cells from the two groups of patients. Interestingly, TN CD34^+^ cells were unaffected by treatment with the selected inflammatory cytokines (either alone or combined) (Fig. 4a). This unresponsive behavior was even more evident when we studied the migratory capacity of the cells (Fig. 4b). Specifically, various combinations of TNF-α, IL-1β, or IL6 significantly stimulated the migration ability of the CD34^+^ cells of *JAK2*^*V617F*^-mutated patients. Notably, no differences were observed between the two groups of patients when spontaneous migration and migration towards CXCL12 alone were tested.

### TN-derived EVs show distinct phenotype, mitochondrial components and pro-survival signals

To elucidate whether crucial factors of the inflammatory network such as EVs have the potential to regulate the CD34^+^ cell behavior of MF, we compared size, phenotype, and mitochondrial content and function of the isolated EVs from TN and *JAK2*^*V627F*^-mutated patients.

No differences were observed when morphology and size of the isolated EVs were analyzed by electron microscopy and nanoparticle tracking analysis (NTA) (Additional file 1: Figure S1a-b).

To systematically evaluate and explore the surface signature of the isolated circulating EVs from patients/HD, we used a multiplex bead-based flow cytometry assay as previously reported [39] (Additional file 1: Figure S2a-c). Exosomal markers such as CD9, CD63, and CD81 were detected at low levels in all isolated EVs. To validate this observation, we repeated these analyses with CD9, CD63- and CD81-antibodies (Additional file 1: Figure S2d).

**Fig. 5:**
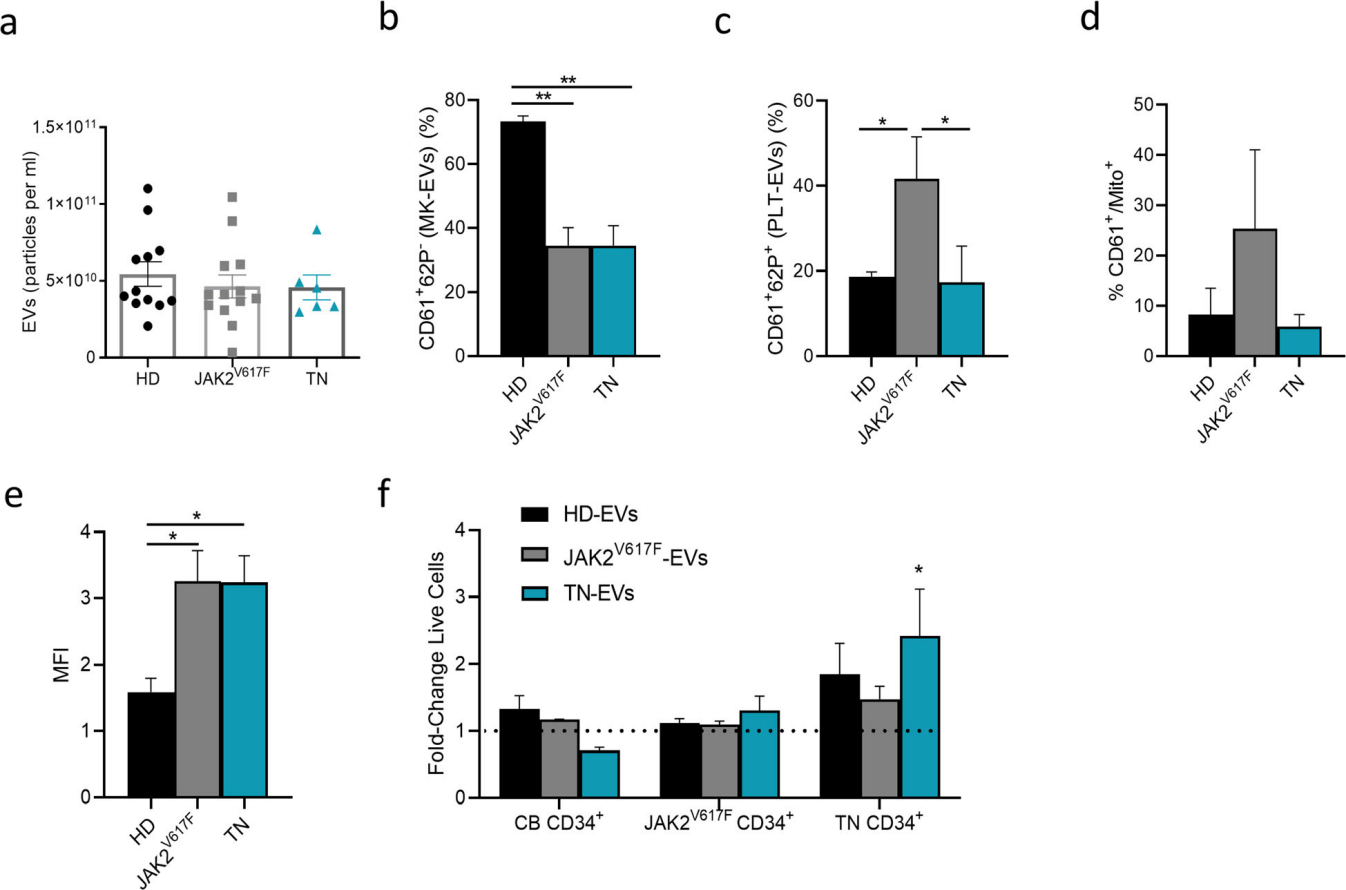
Plasma-derived EV characterization and effects on the survival of MF- or CB-derived CD34^+^ cells. **a** NTA size distribution profiles of the EVs collected from plasma of HD (*n* = 12), *JAK2*^V617F^-mutated (*n* = 13) and TN patients (*n* = 6). **b** Frequency of MK‐EVs (CD61^+^/CD62P^‐^; **c** PLT‐EVs (CD61^+^/CD62P^+^, **d** and Mitotracker-EVs (CD61^+^/Mito^+^); from HD (black, *n* = 3), JAK2^V617F^-mutated (grey, *n* = 3) or TN (blue, *n* = 3) patients. Accordingly, the MitoTracker Red CMXRos MFI in the same samples (**e**). **f** Survival rate as fold-change of cord blood-, *JAK2*^V617F^-, TN-derived CD34^+^ co-cultured with EVs isolated from plasma of HD (black, n=3), *JAK2*^V617F^-mutated (grey, n=3) or TN (blue, *n* = 3). Results are expressed as mean percentages ± SEM (*p ≤ 0.05; **p ≤ 0.01). CB: cord blood

To further characterize the phenotype of the isolated circulating EVs, we specifically analyzed the proportion of the MK- and PLT-EVs by flow cytometry. As shown in Fig. 5b, the MK-EVs were significantly decreased in both patient groups (*p* < 0.01) as compared to the HD counterparts. Conversely, despite comparable platelet counts, the PLT-EVs were significantly increased (*p* < 0.05) in the *JAK2*^*V617F*^-mutated patients compared to both TN patients and HD (*p <* 0.05; Fig. 5c).

We also tested the mitochondria content in plasma EVs. Despite an increase in the percentage of CD61^+^/MitoTracker^+^, EVs from the *JAK2*^*V617F*^-mutated patients (Fig. 5d), the MFI was higher in TN patients only as compared to the HD counterparts (*p* < 0.05, respectively; Fig. 5e). EVs isolated as above were also analyzed by western blot for the presence of the mitochondrial marker TOMM20, a subunit component of the outer mitochondrial membrane. TOMM20 was present in all EVs tested and no differences were reported between the groups at protein levels (Additional file 1: Suppl. S3 a-b).

To analyze the effect of EVs on the in vitro survival of the circulating CD34^+^ cells from patients and normal CD34^+^ from CB, we performed co-culture experiments. Specifically, circulating CD34^+^ cells from CB or *JAK2*^*V617F*^-mutated/TN patients were cultured in the presence of EVs isolated from allogeneic HD, *JAK2*^*V617F*^-mutated, and TN patients, respectively. As shown in Fig. 5f, the survival of CD34^+^ cells from the *JAK2*^*V617F*^-mutated patients was not influenced after co-cultures with any EVs. Conversely, CD34^+^ cells from TN patients were more responsive to EV treatments and showed a significantly increase in survival rate when co-cultured with EVs from TN patients (*p <* 0.05, Fig. 5f).

### The circulating EVs from TN patients are enriched with miR-361-5p

To investigate whether the functional effects of TN EVs might be due to specific cargo, we analyzed the miRNA profile of the isolated EVs from MF patients/HD. After the profiling of 754 miRNAs, those with the highest or lowest fold change were selected for validation (the selected miRs and their FC are reported in Table S3).

**Fig. 6:**
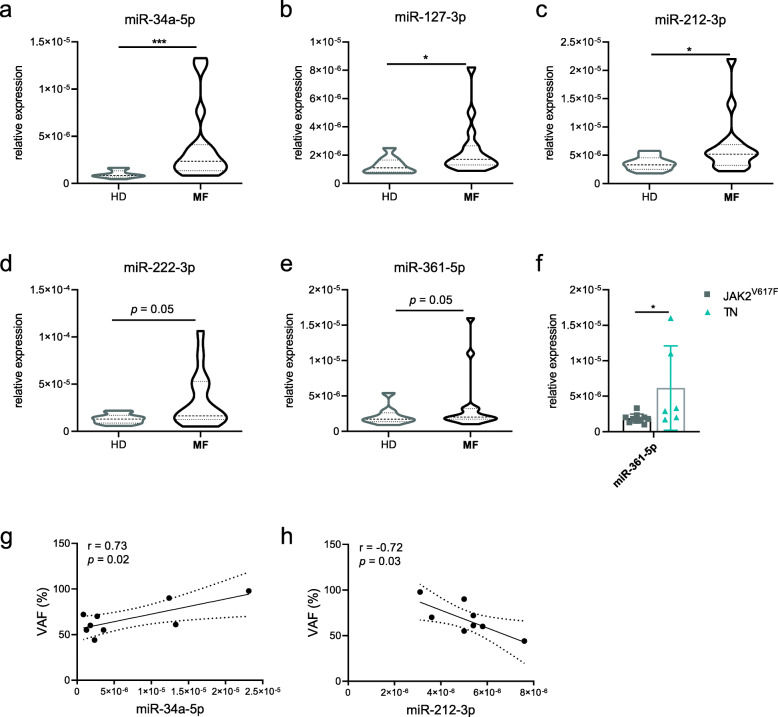
Expression of selected miRNAs in the plasma-derived EVs. Expression of miR-34a-5p (**a**), miR-127-3p (**b**), miR-212-3p (**c**), miR-222-3p (**d**), and miR-361-5p (**e**) in HD-derived EVs (*n* = 10), *JAK2*^*V617F*^-mutated (*n* = 10), and TN (*n* = 6) patients. **f** Expression of miR-361-5p in EVs from *JAK2*^*V617F*^-mutated, and TN patients, respectively. Results are expressed as mean ± SEM (*p ≤ 0.05; **p ≤ 0.01). **g-h** Linear regression analysis showing positive/negative correlation between JAK2^*V617F*^ VAF and miR-34a or miR-212, respectively

Results of the validation analysis by RT-qPCR are shown in Fig. 6a-c. Comparing EVs from MF patients and HD, the expression of 3 miRNAs (miR-34a-5p, − 127-3p, − 212-3p) was significantly upregulated in MF patients. Specifically, miR-34a-5p was up-regulated in both TN (*p =* 0.02) and *JAK2*^*V617F*^-mutated (*p =* 0.005) patients. Conversely, miR-127-3p was significantly up-regulated in *JAK2*^*V617F*^-mutated patients only (*p =* 0.03; data not shown).

Also, miR-222-3p and miR-361-5p showed increased levels in EVs from MF patients (*p =* 0.05, respectively; Fig. 6d-e). The discrepancies between RT-qPCR results and profiling data (in card-arrays) were likely due to the differences of methods and the associated variability of the enlarged cohort of the validation phase. Notably, KEGG analysis (mirPath) of these four miRNAs (miR-34a-5p, − 127-3p, − 212-3p, − 222-3p; genes intersection) revealed PABPC1 (Poly(A) Binding Protein Cytoplasmic 1) as common target gene.

When we compared the miRNA profile of the EVs isolated from the two groups of patients, we observed that only miR-361-5p was differentially expressed and exhibited 3.2-fold higher expression in TN-derived EVs (*p =* 0.04; Fig. 6f). Interestingly, GO category analysis of miR-361-5p showed significant pathways as ‘organelle’, ‘RNA binding’, ‘cellular protein modification process’, and ‘membrane organization’.

mportantly, the *JAK2*^*V617F*^ variant allele frequency (VAF) was positively correlated with miR-34a-5p (r = 0.738; *p =* 0.02), as well as inversely correlated with miR-212-3p (r = − 0.72; *p =* 0.03; Fig. 6g-h).

## Discussion

MF remains an incurable and critical disease [40] and the lack of studies on MF, particularly on the TN subtype, has not conferred advances in the treatment landscape in the last years. In the present study, through an in-depth analysis of HSPCs and key microenvironmental factors including cytokines and EVs, we compared TN and *JAK2*^*V617F*^-mutated patients providing novel insights in the pathogenesis of MF. As clearly reported by us [19] and others [41], the inflammatory microenvironment plays a crucial role in MF pathogenesis; however, few works deserved specific attention to the different molecular subgroups of MF patients.

Firstly, we demonstrated that the in vitro hemopoietic phenotype, survival, and function of circulating CD34^+^ cells are significantly altered in TN patients. Of note, we found an increase in the absolute number of circulating CD34^+^ cells in TN patients as a tool that may predict a more aggressive disease. These data were also consistent with the downregulation reported by GEP in selected genes involved in cell adhesion processes. Therefore, the enumeration of circulating CD34^+^ cells is highly relevant not only for improving diagnosis and risk assessment but also for evaluating response to potentially novel therapeutic approaches.

Specifically, TN patients show ex vivo*/*in vitro increased apoptosis within the CD34^+^ cell compartment with reduced hemopoietic function, revealing a stronger dependence on their microenvironment in comparison to the *JAK2*^*V617F*^-mutated CD34^+^ cells. Indeed, several genes involved in apoptotic and proliferation mechanisms are down-regulated in TN CD34^+^ cells, confirming the presence of deregulated survival pathways and an intrinsic disadvantage of TN CD34^+^ cells in terms of survival and proliferative intracellular signals. Among the differentially expressed genes, we observed an upregulation of tumor protein 53-induced nuclear protein 1 (*TP53INP1*) in TN CD34^+^, which is over-expressed during stress responses including inflammation [42]. *TP53INP1* is a pro-apoptotic gene and its upregulation might partly explain the increased apoptotic rate of the TN CD34^+^ cells compartment in vitro. Consistent with this finding we observed KRAS signature enriched in TN CD34^+^ cells. It is noteworthy that KRAS overexpression confers an adverse prognosis in cytogenetically normal acute myeloid leukemia (AML) [43] and somatic activation of oncogenic KRAS in hematopoietic cells initiates a rapidly fatal myeloproliferative disorder [44]. To sum up, in the present study, the GEP of TN HSPCs might highlight their vulnerability and might explain the more aggressive disease compared with the *JAK2*^*V617F*^ MF molecular subtypes. Furtherly, we previously reported that pro-inflammatory cytokines (e.g. IL1-β, IL6, TNF-α) promoted the survival, clonogenic capacity, and migration of CD34^+^ isolated from MF patients, mostly *JAK2*^V617F^ mutated [19]. By contrast, in the current study, we discovered that CD34^+^ cells from TN patients were insensitive to specific pro-inflammatory cytokines, suggesting their dependence on other factors from their microenvironment. Thus, it remains a matter of discussion whether the defective response to cytokines of TN CD34^+^ cells is related to altered apoptosis pathway, as reported by GEP analysis, “exhaustion” driven by previous inflammatory status, or to the dependence on “to be defined” factors from the microenvironment.

Notably, comparing the cytokine profiling between two subtypes of patients, TN patients show a milder inflammatory phenotype suggesting that therapies targeting the cytokine storm might be ineffective in this subset of MF patients. Moreover, only TPO plasma levels were lower in TN patients compared to the *JAK2*^*V617F*^-mutated patients, suggesting TPO as a biomarker of poor prognosis in MF. In accordance, *Seiki Y* et al. [45] reported relatively low TPO levels in Myelodysplastic syndromes (MDS) patients and that low levels of TPO were associated with poor prognosis and progression to AML. For the first time, we also reported higher TPO levels in female MF compared to males. Interestingly, *Barraco* et al. [46] observed that female MF patients had a better prognosis with slower disease progression. Consequently, all these data point out that an inflammatory microenvironment is closely associated with the *JAK2*^*V617F*^ mutation but not to the TN counterparts. Whether this is due to the presence of a normally activated JAK/STAT pathway in TN patients remains a matter of speculation.

Based on our in vitro results, we then sought to determine whether the functional behavior of TN CD34^+^ cells might be influenced by circulating EVs as signals from the microenvironment. Here, for the first time, we analyzed the phenotype, the mitochondrial content, and the miRNA cargo profile of the isolated circulating EVs from MF patients/HD. Since most circulating EVs are of megakaryocyte and platelet origin, we demonstrated that the MK-EVs were significantly reduced in both *JAK2*^*V617F*^-mutated and TN patients. This finding confirms the occurrence of megakaryocyte abnormalities in MF; conversely, the PLT-EVs were decreased in TN patients. Of note, a previous study demonstrated that TPO may promote platelet aggregation [47]; therefore, it is likely that the increased level of circulating TPO in the *JAK2*^*V617F*^-mutated patients may partly contribute to the platelet activation and, as a consequence, to PLT-EVs release.

Recently, it has been described the existence and function of intact mitochondria or mitochondrial constituents transferred through EVs [48]. Consistently, platelet EVs may transport mitochondria as a mechanism to mediate inflammation [49] or immune cell regulation [28]. Conversely, mitochondria, either naked or encapsulated by EVs, may trigger inflammation, may interact with innate immune signaling pathways and may regulate cell metabolism, apoptosis, and various pathophysiological situations [50]. Here, we observed the presence of mitochondrial outer membrane protein TOMM20 in EVs from plasma and, interestingly, EVs from MF patients carry the highest level of MitoTracker MFI when compared with the healthy counterparts. These results, suggesting the presence of mitochondria in the EVs of our study, might be also in contrast to the TEM analysis that did not detect any intact mitochondria. However, although MitoTracker Red has been utilized as a membrane potential-sensitive dye, mitochondrial staining has several limitations [51, 52] and up to date has not been fully explored in EVs. Therefore, our data support the hypothesis that the MitoTracker dye might be associated with mitochondrial components rather than with respiring mitochondria in EVs. Despite the need for further studies, this finding might play a role in the Darwinian selection of cancer cells with the potential of fueling tumor cell maintenance and proliferation. As far as the increased prevalence in elderly patients is concerned, the role of inflammaging, i.e. the low chronic level of the inflammatory status associated with the aging process [20], could be another important risk factor for the change of microenvironment, making the cells more susceptible to undergo transformation, particularly for *JAK2*^*V617F*^-mutated patients. In this perspective, the additional role of mitochondria circulation, a phenomenon likely related to the PB increase of mtDNA with aging [53], and their possible transfer into other cells should be further addressed. To our knowledge, the mitochondria content of EVs is for the first time reported in MF patients and this might represent a novel clinically relevant therapeutic target that needs further investigations.

Recently, it has been also described that the miRNA cargo of EVs might have a prognostic role and can be used to evaluate disease progression [54, 55]. Comparing MF patients and HD, we showed that a distinct miRNA profiling characterizes the MF patients EVs with upregulation of miR-34a-5p, − 127-3p, and − 212-3p. In accordance, *Bianchi* et al. [56] found miR-34a-5p upregulation in PMF CD34^+^ hematopoietic progenitor cells, demonstrating that its overexpression favors the megakaryocyte and monocyte commitment of CD34^+^ cells. Therefore, our data suggest that the alteration in MF megakaryocytes might be also due to EV-driven signals and not only to intrinsic mechanisms encountered in CD34^+^ cells. It is therefore not surprising that miR-34a-5p expression is positively associated with the *JAK2*^*V617F*^ allele burden. Of note, miR-212-3p expression was negatively associated with the *JAK2*^*V617F*^ allele burden, in line with its role as tumor suppressor observed in AML [57]. Additionally, we also confirmed the upregulation of miR-127-3p, another miR highly expressed in MF CD34^+^ from MF patients as previously reported [58]. Specifically, we observed a high miR-127-3p expression in the EVs isolated from the *JAK2*^*V617F*^-mutated MF patients. Similarly to miR-34a [59], miR-127 has been associated with DNA damage response and with cellular senescence [60].

Notably, the common target of the identified miRNAs, i.e. PABPC1, plays a central role in mRNA processing binding the poly(A) tail of mRNA. PABPC1 deregulation by miRNAs interaction could contribute to making susceptible the cells of the microenvironment/niche to MF pathogenesis being known its role in carcinogenesis [61]. Thus, it is interesting the hypothesis of potential epigenetic modulators which, circulating in the blood via EVs, may also transport “potential cancer signals” far from the original site of cell development, accordingly to the theory of “inflammaging and garb-aging” [62].

Importantly, comparing the two groups of patients, only miR-361-5p expression was significantly upregulated in the TN EVs. Previous studies produced somewhat controversial results related to miR-361 [63]. Increased expression of miR-361 was detected in AML suggesting that miR-361 dysregulation might be required to impair differentiation program in hemopoiesis and leukemia [63]. It has also become apparent that miR-361 can downregulate the mRNA expression of IL-6 and IL-8 in endometrial cancer cells through targeting TWIST [64]. This finding suggests a potential anti-inflammatory role of miR-361-5p, which is in line with the mild pro-inflammatory profile observed in TN patients.

## Conclusions

In conclusion, although the number of our patients is small and the study involved a rare MF subclass, the data presented here provide further insights into the pathogenesis of MF and highlight the biomolecular profile of TN CD34^+^ cells and circulating EVs within the inflammaging conceptualization. Our data demonstrate that in TN MF intrinsic HSPCs defects are associated with an impaired response to microenvironment signals. The results of this study may have diagnostic and prognostic implications in MF and suggest that TN patients deserve a personalized therapeutic approach targeting not only the stem cell compartment but also the EVs. Despite the specific role of EVs and the changes in their cargo remain to be further elucidated, we revealed crucial biomolecular vulnerabilities of TN patients providing the basis for novel and unexplored druggable pathways.

## Supplementary Information

**Additional file 1: Table S1.** Characteristics of the patient population. **Table S2.** Differentially expressed transcripts. **Table S3.** List of the selected miRNAs with their relative fold-changes (FC). **Figure S1.** Characterization by TME and NTA of EVs. **Figure S2.** EV characterization by flow cytometry. **Figure S3.** Representative western blot and quantification graph for TOMM20.

## Data Availability

The datasets used and analyzed during the current study are available upon reasonable request.

## Abbreviations

AML: Acute myeloid leukemia
BFU-E: Burst Forming Unit-erythroid
CB: Cord blood
CFU-C: Colony-forming capacity
CFU-GM: Colony forming unit-granulocyte macrophage
EVs: Extracellular vesicles
GEP: Gene expression profiling
HD: Healthy donors
HSPCs: Hemopoietic stem/progenitor cells
IL: Interleukin
MDS: Myelodysplastic syndromes
MF: Myelofibrosis
MFI: Mean fluorescence intensity
miRNA: microRNAs
MK: Megakaryocyte
MNC: Mononuclear cells
MPN: Myeloproliferative neoplasm
NTA: Nanoparticle tracking analysis
PABPC1: Poly(A) Binding Protein Cytoplasmic 1
PB: Peripheral blood
PET: Post essential thrombocythemia
PLT: Platelets
PMF: Primary myelofibrosis
PPP: Platelet-poor plasma
PPV: Post polycythemia vera
SMF: Secondary myelofibrosis
TEM: Transmission electron microscopy
TN: Triple-negative
TNF: Tumor necrosis factor
WB: Western blot
