# Supplementary material for: Distinct profile of CD34+ cells and plasma-derived extracellular vesicles from triple-negative patients with Myelofibrosis reveals potential markers of aggressive disease
Source: J Exp Clin Cancer Res. 2021 Feb 1;40:49. doi: 10.1186/s13046-020-01776-8 (PMC7849077; doi:10.1186/s13046-020-01776-8)
Supplement: Supplementary file 1 — Additional file 1: Table S1. Characteristics of the patient population. Table S2. Differentially expressed transcripts. Table S3. List of the selected miRNAs with their relative fold-changes (FC). Figure S1. Characterization by TME and NTA of EVs. Figure S2. EV characterization by flow cytometry. Figure S3. Representative western blot and quantification graph for TOMM20. [file 13046_2020_1776_MOESM1_ESM.pdf]

Supplementary materials

Tables

Table S1. Characteristics of the patient population.

	**Total MF (29 cases)**	**$JAK2^{V617F}$ (23 cases)**	**TN (6 cases)**
Median age, years (range)	72 (43-84)	71 (43-82)	75 (60-84)
Males, no. (%)	16 (55%)	13 (52%)	3 (50%)
Median Hemoglobin, g/dl; median (range)	11.3 (7.1-17.2)	11.5 (7.1-17.2)	9.7 (7.5-14.3)
Median Leukocytes, x 10^9/l; median (range)	10.5 (2.5-40)	10.5 (4.7-40)	10.7 (2.5-27)
Median Platelets, x 10^9/l; median (range)	168 (38-631)	174 (48-631)	121 (38-613)
Median Lymphocyte x 10^9/l; median (range)	1.2 (0.2-11.7)	1.1 (0.2-5.6)	4.1 (0.4 -11.7)*
Median Monocyte x 10^9/l; median (range)	0.5 (0.05-5)	0.4 (0.05-2)	0.7 (0.2-5.3)
Blood blasts ≥1%	8 (27%)	6 (26%)	2 (33%)
TSS >20	3 (10%)	3 (13%)	0
Spleen ≥10 cm	13 (44%)	11 (47%)	2 (33%)
**BM fibrosis, no. of patients (%)**			
Grade 2	22 (75.8 %)	16 (69.6%)	6 (100%)
Grade 3	7 (24%)	7 (30%)	0
**IPSS, no. of patients (%)**			
Low	2 (6%)	2 (8%)	0
Intermediate-1	11 (37%)	8 (34%)	3 (50%)
Intermediate-2	7 (24%)	6 (26%)	1 (17%)
High	9 (31%)	7 (30%)	2 (33%)
**WHO Diagnosis**			
PMF	17 (58%)	12 (52%)	5 (83%)
PPV-MF	10 (34%)	10 (43%)	0
PET-MF	2 (6%)	1 (4%)	1 (17%)
Unfavourable karyotype [1]	11	10	1

[1] by DIPPS-plus

Table S2. Pathway enrichment analysis for differentially expressed transcripts between EVs from TN and *JAK2V617F*-mutated patients.

Source	Pathway	P value	Genes
GO-BP	Antigen processing and presentation	2.22E-07	HLA-DQB1, HLA-DRB1, ULBP3, HLA-DRB4, HLA-B, HLA-DQA2, CD74, HLA-DQA1
KEGG	Antigen processing and presentation	6.55E-05	HLA-DQB1, HLA-DRB1, HLA-DRB4, HLA-B, HLA-DQA2, CD74, HLA-DQA1
GO-BP	Interferon-gamma-mediated signaling pathway	2.31E-04	HLA-DQB1, HLA-DRB1, HLA-DRB4, HLA-B, HLA-DQA2, HLA-DQA1
KEGG	Cell adhesion molecules (CAMs)	3.12E-04	HLA-DQB1, ITGAL, HLA-DRB1, HLA-DRB4, CNTNAP2, HLA-B, HLA-DQA2, HLA-DQA1
GO-BP	Immune response	4.63E-04	HLA-DQB1, TNFRSF1B, TNFSF10, TNFSF4, HLA-DRB1, HRH2, HLA-DRB4, HLA-B, HLA-DQA2, CD74, HLA-DQA1, CTSG
GO-BP	Regulation of immune response	0.0129	ITGAL, ULBP3, TREML2, HLA-B, TYROBP, HCST
GO-BP	Calcium-dependent cell-cell adhesion via plasma membrane cell adhesion molecules	0.0200	PCDHB9, PCDHB14, PCDHB11
GO-BP	Homophilic cell adhesion via plasma membrane adhesion molecules	0.0353	PCDHB9, ROBO1, PCDHB14, PCDHB12, PCDHB11

GO-BP, gene ontology biological process

Table S3. List of the selected miRNAs with their relative fold-changes (FC).

MF vs HD	
microRNA (miR)	FC
miR-127-3p	9.25
miR-15b-5p	6.69
miR-221-3p	5.62
miR-19a-3p	4.07
miR-21-5p	2.53
miR-146a-5p	2.27
miR-222-3p	2.18
miR-24-3p	2.07
miR-155-5p	2.01
miR-202-3p	-2.27
miR-212-3p	-2.60

TN vs JAK2^{V617F}	
microRNA (miR)	FC
miR-122-5p	2.55
miR-365a-3p	2.52
miR-744-5p	2.47
miR-27a-3p	2.32
miR-548c-3p	2.05
miR-361-5p	-2.23
let-7b-5p	-2.45
miR-34a-5p	-4.50

MiRs validated by RT-qPCR are reported in red.

Figures

Figure S1. EV characterization by TME and NTA

a

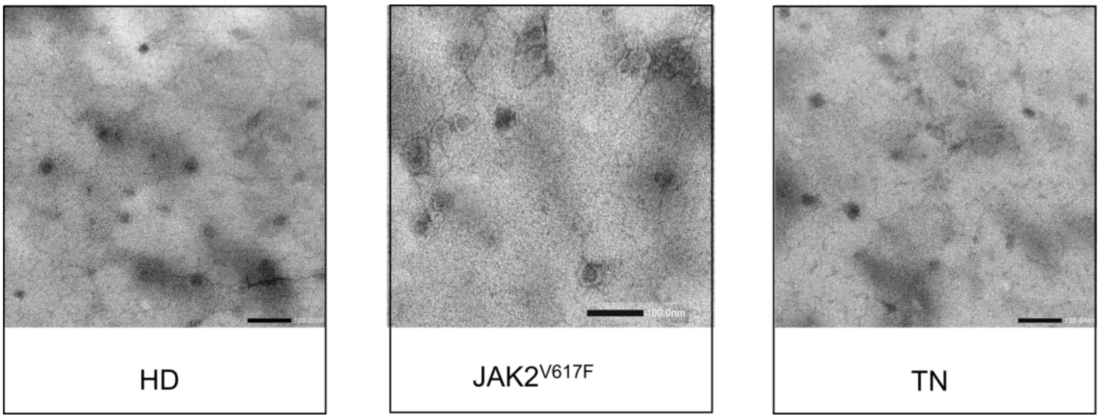

b

Results	HD	JAK2^{V617F}	TN
Mean	116 ± 13.4	119 ± 14.7	125.2 ± 14.1
Mode	78.7 ± 13.4	85.4 ± 14.4	89.74 ± 19
SD	52.8 ± 8.5	53.6 ± 9.1	52.34 ± 4.5
D10	67.7 ± 8.6	67.8 ± 8.5	74.61 ± 14
D50	101.2 ± 10.8	104.8 ± 13.6	111 ± 13.9
D90	183.1 ± 24.6	185 ± 25.1	189.6 ± 16.3

Figure S1. Characterization of EVs isolated from plasma by ultracentrifugation. (a) Whole-mount transmission electron micrograph of EVs displaying the characteristic EV morphology in each sample between HD, *JAK2V617F*-mutated and TN patients. b) Summary of data obtained by Nanosight equipment for each EV isolated by ultracentrifugation.

Figure S2. Flow cytometry characterization of EVs.

a

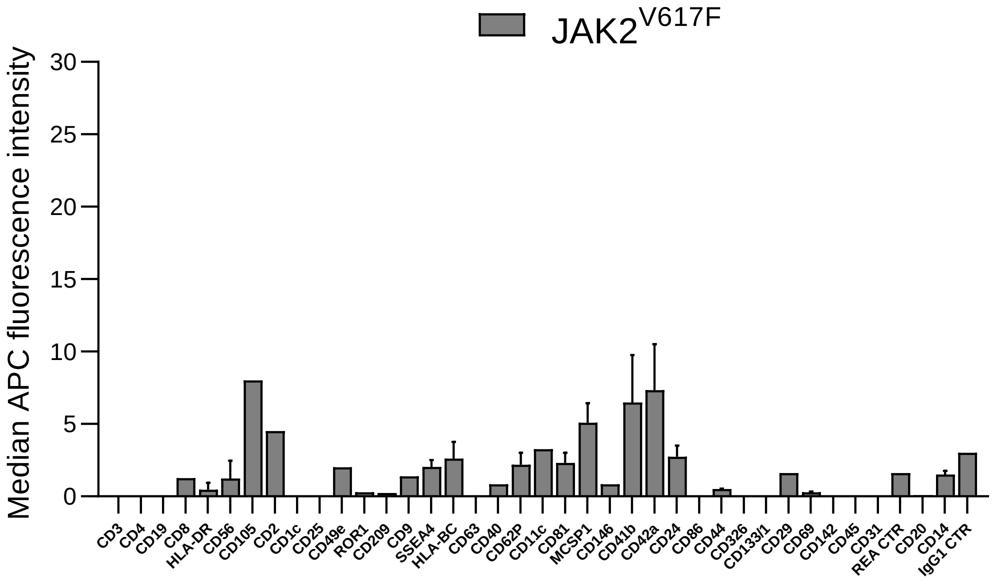

b

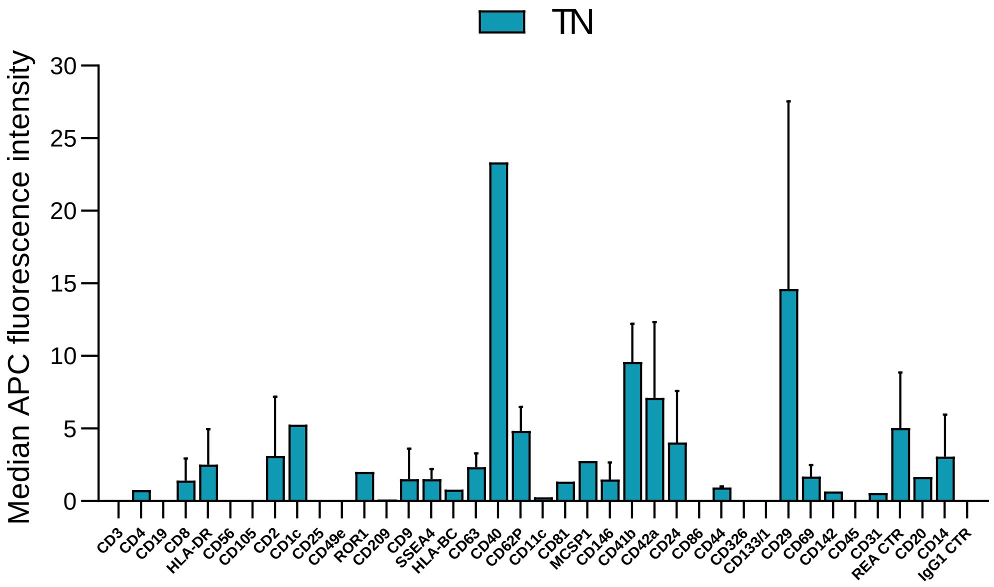

c

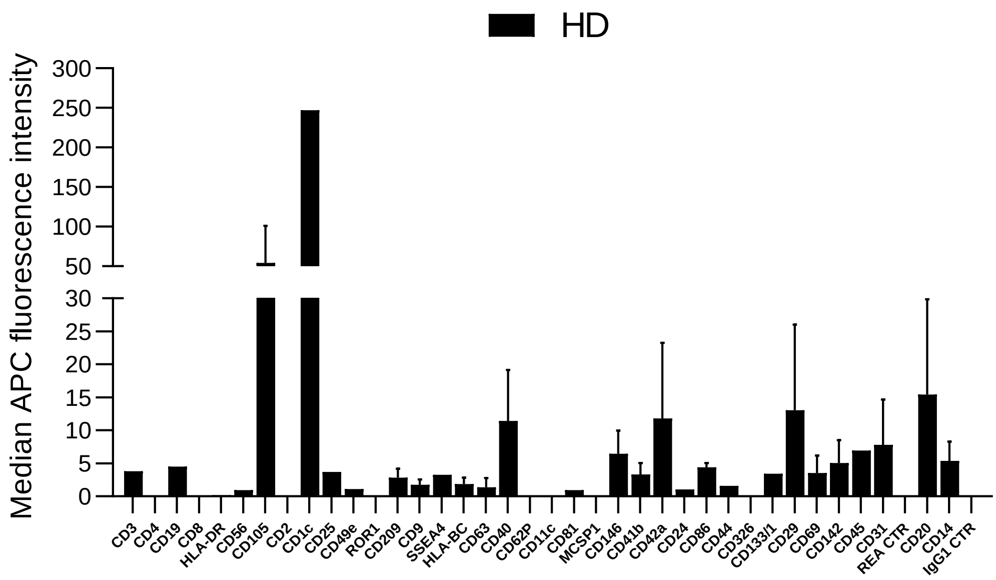

d

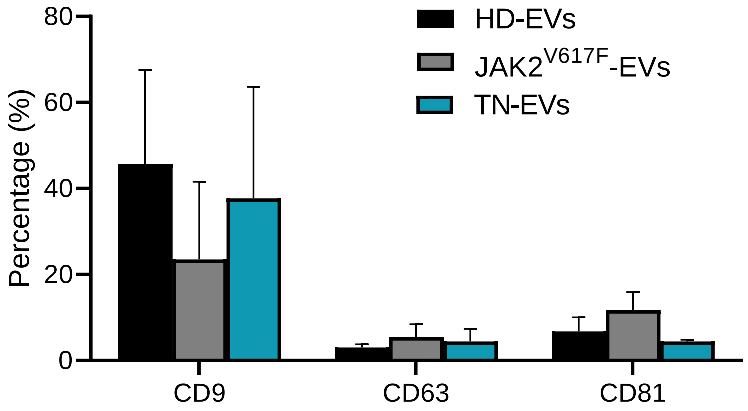

Figure S2. Surface marker profiles of plasma-derived EV from MF patients and HD. Background corrected APC median signal intensities after incubation of plasma-derived EVs from 3 JAK2^{V617F}-mutated patients (a) or 3 TN patients (b) and 3 HD (c). EVs from plasma with 39 capture antibody bead populations, followed by staining with a cocktail of anti-CD9-, anti-CD63- and anti-CD81-APC antibodies. REA, mIgG1 indicate isotype control-beads. d) CD9, CD63, CD81 frequency in EVs by flow cytometry.

Figure S3. Representative western blot and quantification graph for TOMM20

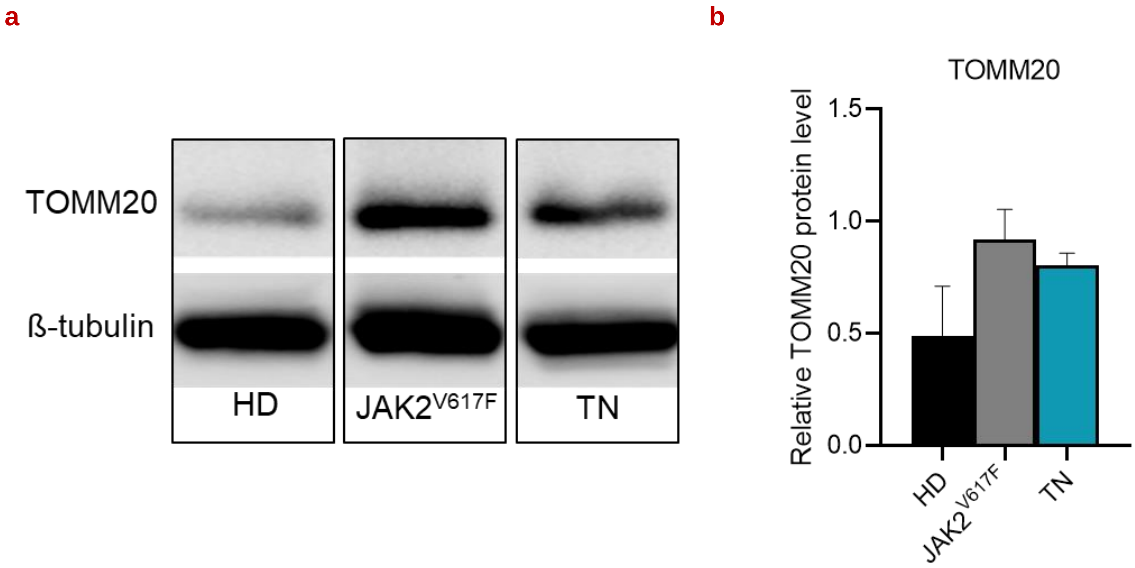

a) Representative western blot of EVs preparation from HD, JAK2^{V617F}-mutated, and TN patient-derived plasma. β-tubulin was used as a loading control. b) Graphic representation of quantification for TOMM20 expression normalized to the relative β-tubulin from HD (n=3), JAK2^{V617F}-mutated (n=3), and TN (n=2) patients. The translocase of outer mitochondrial membrane 20 (TOMM20) protein level was determined using the ImageJ analysis software and normalized to β-tubulin, taken as an index of loading control. Histograms show the mean ± SEM.
